# The clinical characteristics of adults with rheumatic heart disease in Yangon, Myanmar: An observational study

**DOI:** 10.1371/journal.pone.0192880

**Published:** 2018-02-21

**Authors:** Nan Phyu Sin Toe Myint, Ne Myo Aung, Myint Soe Win, Thu Ya Htut, Anna P. Ralph, David A. Cooper, Myo Lwin Nyein, Mar Mar Kyi, Josh Hanson

**Affiliations:** 1 Department of Medicine, Insein General Hospital, Yangon, Myanmar; 2 University of Medicine 2, Yangon, Myanmar; 3 Department of Cardiology, North Okkalapa General Hospital, Yangon, Myanmar; 4 Global and Tropical Health Division, Menzies School of Health Research, Charles Darwin University, Darwin, Australia; 5 Director’s Unit, Kirby Institute, University of New South Wales, Sydney, Australia; Osaka University Graduate School of Medicine, JAPAN

## Abstract

**Background:**

Rheumatic heart disease (RHD) is a major cause of premature death in low and middle-income countries. The greatest barrier to RHD control is neglect of the disease in national health policies and a lack of prevalence data that might inform control efforts. Myanmar is making remarkable progress against many infectious diseases, but there are almost no data to define the clinical burden of RHD in the country. This prospective audit was performed in an adult medical ward of a tertiary-referral hospital in Yangon, to gain an insight into the prevalence of RHD in Myanmar.

**Principal findings:**

All patients admitted to the ward between May 1, 2016 and April 30, 2017 were eligible for enrolment. RHD was confirmed in 96 patients who were admitted on 134 occasions, representing 1.1% of the 12,172 adult medical admissions during the study period. This compared with 410 (3.4%) admissions with HIV and 14 (0.1%) with malaria. Patients with RHD had a median age of 44 years (interquartile range: 35–59); 70 (73%) were female. Only one patient had ever had surgery despite 79 (82%) meeting criteria for intervention; 54 (56%) patients were not receiving any regular clinician review. Prior to hospitalisation only 18 (19%) patients were receiving regular penicillin. Only 8 (19%) of the 42 women <50 years were using contraception. Of 49 patients who had been hospitalised previously, 22 (45%) were receiving no regular therapy. During the study three (3.1%) patients died, and 28 (29%) were lost to follow-up. Of the 65 (68%) alive and retained in care, 21 (32%) were still experiencing moderate-severe RHD-related symptoms at the study’s end.

**Conclusions:**

There is a significant and unmet clinical burden of RHD in Myanmar. A national RHD programme would improve patient care, reducing morbidity and mortality from this preventable disease.

## Background

Rheumatic heart disease (RHD) is preventable, but globally in 2015 it was responsible for almost 320,000 deaths and the loss of 10.5 million disability-adjusted life years [1]. Incomplete data collection in the low and middle-income countries most affected by RHD means that its burden may be even greater [2, 3]. Underestimating the prevalence of RHD leads to its neglect in national health policies and is one of greatest barriers to countering the disease [4].

Myanmar is at a critical time in its history and its newly elected government faces enormous challenges in improving the health of its people [5, 6]. In some areas, there has been significant progress. The Global Fund to treat AIDS, Tuberculosis and Malaria (GFATM) has provided over USD 500 million in financial support to Myanmar and this has led to some remarkable gains [7]. In the last decade, deaths from malaria in Myanmar have fallen by up to 90%, while deaths related to human immunodeficiency virus (HIV) and tuberculosis have fallen by over 50% [8, 9]. While the financial support of the GFATM has been essential for this success, the fact that Myanmar has a government sponsored national programme for the control of all three diseases has permitted targeted, coordinated and effective deployment of the available resources [8, 10, 11].

However, despite a recognition among practicing clinicians that RHD is common in Myanmar, the disease’s clinical burden in the country is poorly defined [12–15] and efforts to address the disease lack the systematic and multifaceted approach that is being taken in the national programmes to control malaria, HIV and tuberculosis.

This clinical audit was performed to provide a detailed description of the clinical characteristics and presentation of adults with RHD in Myanmar, as well as the challenges of recognising and treating the condition in the country’s under-resourced public health system. By defining the clinical burden of RHD, the study also had the goal of raising awareness of the disease in Myanmar and to provide support for the development of a national response.

## Methods

The study was performed between May 1, 2016 and April 30, 2017 at Insein General Hospital, a university-affiliated, tertiary referral hospital in Yangon, the largest city in Myanmar. The hospital serves Insein Township which has a population of 305,283 people, including 240,175 people aged 15 years or over [16].

Every patient admitted to the adult medical ward of the hospital during the study period was eligible for inclusion. Patients were assessed on admission to hospital and were enrolled in the study if there were clinical signs of RHD (an audible murmur, new atrial fibrillation or new pulmonary congestion) on examination. Study doctors then reviewed the patient’s prior medical record, collected a focussed medical history using a dedicated *pro forma*, performed a physical examination and requested an electrocardiogram (ECG), a chest x-ray (CXR) and an echocardiogram. There are no on-site echocardiography services at Insein General Hospital and so patients were referred to the cardiology department at North Okkalapa General Hospital, the nearest public service, 7.6 kilometres away. The presence of RHD was defined using World Heart Federation criteria for echocardiographic diagnosis of rheumatic heart disease [17] and a requirement for surgery was defined using European Society of Cardiology criteria [18]. Suitability for percutaneous balloon mitral valvulotomy (PBMV) was determined using the Wilkins score [19]. Patients enrolled in the study were followed up in the medical outpatients and by telephone if the patients failed to attend outpatients.

Data were recorded in an electronic database (S1 Dataset) and were analysed using statistical software (Stata 14.0).

Ethical approval for the study was granted by the Human Research Ethics Committees of the University of Medicine 2, Yangon and the Menzies School of Health Research, Darwin, Australia. As the study was purely observational, both committees waived the requirement for informed consent.

## Results

**Fig 1:**
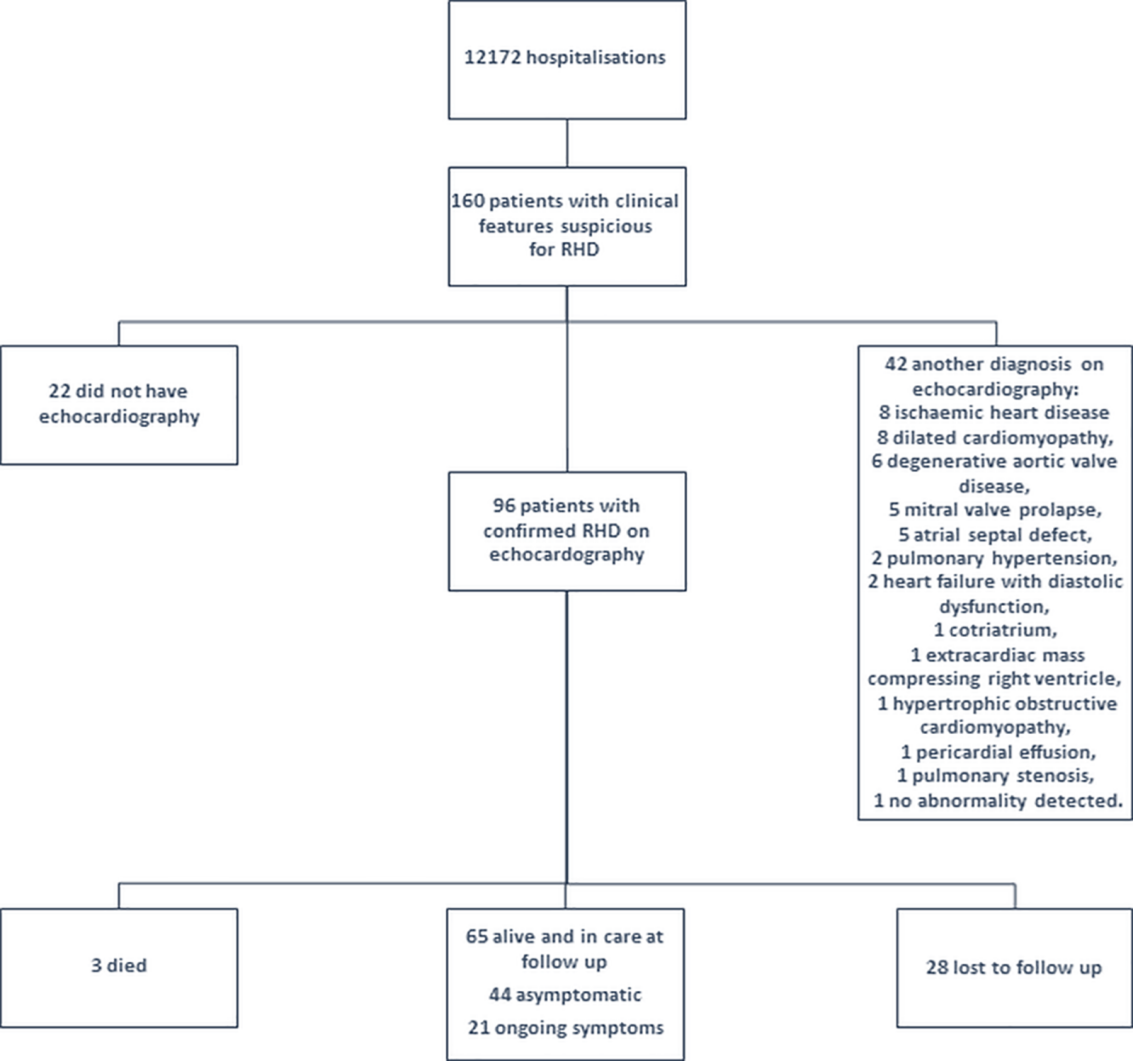
Cohort diagram showing screening, enrolment and the results of follow-up of the cohort.

During the twelve months of the study period, there were 160 patients admitted to the adult medical ward with clinical signs suspicious for RHD. There were 96 (60%) patients with echocardiographically-confirmed RHD, 22 (14%) patients were not able to receive an echocardiogram, while 42 (26%) had an echocardiogram which suggested a diagnosis other than RHD (Fig 1). Only the 96 patients with confirmed RHD were included in the analysis. Their baseline characteristics are presented in Table 1.

**Table 1:** Baseline characteristics of the cohort.

Characteristics of the cohort at enrolment	
Female (number, %)	70 (73%)
Age (years)	44 (35–59)
History of ARF	40 (42%)
Any ARF treatment	40 (42%)
Complete ARF treatment ^a^	3 (3%)
Prior echocardiogram	12 (13%)
Prior hospitalisation for RHD symptoms	49 (51%)
Prior stroke	11(11%)
Prior surgical intervention	1 (1%)
Prior pregnancy	56/70 (80%)
Prior hospitalisation for RHD symptoms during pregnancy	8/70 (14%)

All values represent number (%) or median (interquartile range)

ARF: acute rheumatic fever; RHD: rheumatic heart disease

^a^ Echocardiogram, penicillin prophylaxis and at least annual review.

**Fig 2:**
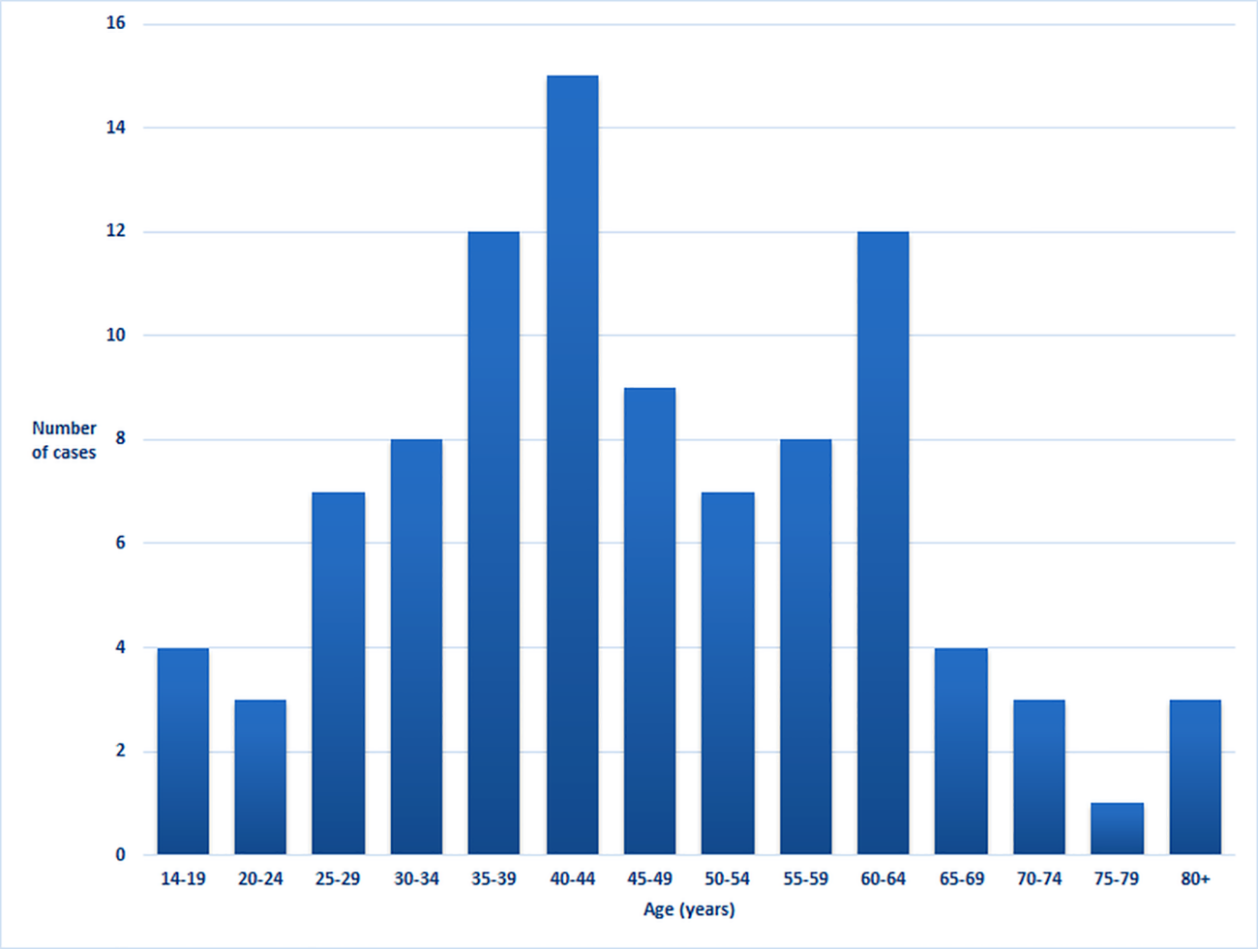
Age of patients in the cohort on enrolment.

The 96 patients were admitted on 134 occasions, representing 1.1% of the 12172 adult medical admissions during the study period. This compared with 975 (8%) patient admissions with tuberculosis, 410 (3.4%) patient admissions with HIV-related illness and 14 (0.1%) patient admissions with malaria over the same time. The RHD patients’ median age was 44 years (interquartile range: 35–59, range: 14–82) (Fig 2); 70 (73%) were female.

### History

Forty (42%) patients had a history compatible with a diagnosis of past acute rheumatic fever (ARF), and of these, 21 (53%) had a history consistent with recurrent ARF, although with limited access to echocardiography and laboratory testing, no patient had a confirmed diagnosis satisfying Jones’ criteria [20]. Indeed, only eight (20%) of these 40 patients had received a previous echocardiogram and in only four (10%) were the echocardiogram findings documented in their medical record.

Of the 96 patients, 49 (51%) had been hospitalised for RHD-related symptoms previously; eleven (11%) had had a previous stroke, three (27%) of whom had continuing severe disability. Of the 70 women, 56 (80%) had been pregnant, 8 (14%) of whom had required hospitalisation during pregnancy. There were 79 (82%) patients with daily symptoms from their RHD; the commonest symptom was shortness of breath on exertion, which was present in 72 (91%) of those with daily symptoms. In 41 (57%) this was New York Heart Association class III or greater. A single patient had received surgical intervention in the form of mitral valve repair.

### Access to regular medical review

Among the cohort, 42 (44%) patients were receiving regular review by a general practitioner and 31 (32%) were receiving regular specialist review. Only three (3%) patients had regular cardiology review and only one (1%) had seen a cardiothoracic surgeon. There were 54 (56%) patients who were not receiving any regular clinician review.

### Medication prior to hospitalisation

Prior to hospitalisation 18 (19%) patients were receiving regular penicillin as ARF prophylaxis (13 orally, 5 intramuscularly); this included 11/62 (17%) patients ≥40, 7/34 (21%) of patients <40 years of age and 12/40 (30%) patients with a history of ARF.

Nine patients were receiving anticoagulation—all warfarin—all of whom were having international normalised ratio (INR) monitoring at least monthly. However, only two (18%) of the eleven patients who had previously had a stroke were anticoagulated. There were 23 (24%) patients receiving an agent for rate control of atrial fibrillation, although only six (26%) of these 23 were also receiving warfarin. Of the 49 patients previously hospitalised with symptoms related to RHD, 22 (45%) were not receiving any regular medical therapy for the condition.

Only 8 (19%) of the 42 women younger than 50 years of age were using contraception; 4 of 5 of the women of child bearing age who had previously been hospitalised during pregnancy were not using contraception.

### Clinical presentation

Of the 96 patients, 88 (92%) had cardiovascular symptoms on presentation (dyspnoea, palpitations, peripheral oedema or syncope). The commonest reasons for admission were atrial fibrillation with a rapid ventricular response (29 cases (30%)) and pulmonary congestion (28 cases (29%)). There were eight (8%) patients admitted with a stroke, only one (13%) of whom was receiving anticoagulation. One patient (1%) was admitted with infective endocarditis. Five (7%) women were pregnant on admission. Non-cardiovascular diagnoses on admission included pneumonia and exacerbation of chronic obstructive pulmonary disease. On admission, 60 (63%) patients were in atrial fibrillation or atrial flutter, 30 (50%) of whom were tachycardic (heart rate >100 beats per minute). There were 50 (52%) patients with clinical evidence of pulmonary congestion (crepitations on auscultation), 6 (6%) of whom had an oxygen saturation on room air of less than 90%). Most patients (54 (56%)) had poor dentition. The other findings on physical examination are presented in Table 2.

**Table 2:** Physical examination findings.

Physical examination finding	
Systolic blood pressure (mmHg)	110 (100–130)
Diastolic blood pressure (mmHg)	70 (60–90)
Heart rate (beats per minute)	98 (79–116)
Respiratory rate (breaths per minute)	18 (16–20)
Oxygen saturation on room air (%)	97 (95–98)
Body mass index kg/m^2^	20.5 (18.4–22.1)
Atrial fibrillation	55 (57%)
Elevated jugular venous pressure	29 (30%)
Signs of pulmonary hypertension	56 (58%)
Both systolic and diastolic murmur	33 (34%)
Only systolic murmur	21 (22%)
Only diastolic murmur	42 (44%)
Signs of pulmonary congestion	50 (52%)
Hepatomegaly	16 (17%)
Ascites	3 (3%)
Peripheral oedema	15 (16%)
Poor dentition	54 (56%)

All values represent number (%) or median (interquartile range)

mmHg: millimetres of mercury

### Electrocardiography findings

All but 2 of the 96 patients had an abnormal electrocardiogram (ECG). The most common abnormalities were right axis deviation (present in 61 (64%)) and atrial fibrillation (present in 59 (61%)); one patient was in atrial flutter). The other ECG findings are presented in Table 3.

**Table 3:** Findings of clinical investigations on enrolment.

ECG	Number (%)
Normal	2 (2%)
Left axis deviation	12 (13%)
Right axis deviation	61 (63%)
Atrial fibrillation or flutter	60 (63%)
Right atrial enlargement	8 (8%)
Left atrial enlargement	25 (26%)
RBBB	7 (7%)
LBBB	0
Right heart strain/RVH	28 (29%)
Left heart strain/LVH	32 (33%)
CXR	
Normal	5 (5%)
Increased cardiothoracic ratio	83/92
Left atrial enlargement ^a^	64 (67%)
Pulmonary hypertension ^b^	64 (67%)
Pulmonary congestion ^c^	23 (24%)
Pleural effusion	1 (1%)
Echocardiogram	
Rheumatic mitral valve	92 (96%)
Rheumatic aortic valve	42 (44%)
Rheumatic pulmonary valve	0
Rheumatic tricuspid valve	0
Left atrial enlargement	79 (82%)
Left atrial diameter (mm)	51 (41–59)
Left ventricular ejection fraction (%)	58 (48–65)
Dilated right ventricle	20/67 (30%)
TAPSE (mm)	16 (14–22)
LVIDd (mm)	47 (43–54)
LVIDs (mm)	33 (29–38)
Estimated PASP (mmHg)	50 (37–65)
PHT likely ^d^	42/76 (55%)
Mitral stenosis	71 (74%)
Severe mitral stenosis ^e^	51 (53%)
Mitral regurgitation	79 (82%)
Severe mitral regurgitation ^e^	21 (22%)
Aortic stenosis	17 (18%)
Severe aortic stenosis ^e^	3 (3%)
Aortic regurgitation	55 (57%)
Severe aortic regurgitation ^e^	9 (9%)
Mobile vegetation	1 (1%)

ECG: electrocardiogram; CXR: chest x-ray; LBBB: left bundle branch block; RBBB: right bundle branch block; RVH: right ventricular hypertrophy LVH: left ventricular hypertrophy TAPSE: tricuspid annular systolic excursion; LVIDd: left ventricular internal diameter (diastole); LVIDs: left ventricular internal diameter (systole); mmHg: millimetres of mercury; PASP: pulmonary artery systolic pressure; PHT: pulmonary hypertension

^a^ Straightened left heart border, double atrial shadow, splayed carina

^b^ Prominent pulmonary arteries with peripheral pruning

^c^ Upper lobe diversion, Kerley B lines, Oedema (alveolar shadowing)

^d^ Estimated PASP≥50mmHg

^e^ European Society of Cardiology criteria [18]

### Chest X-ray findings

All 96 patients had a CXR performed, in all but 5 (95%) it was abnormal. The most common abnormalities were an increased cardio-thoracic ratio (present in 86 (90%)) and evidence of left atrial enlargement (present in 64 (67%)). The other CXR findings are presented in Table 3.

### Echocardiography findings

**Fig 3:**
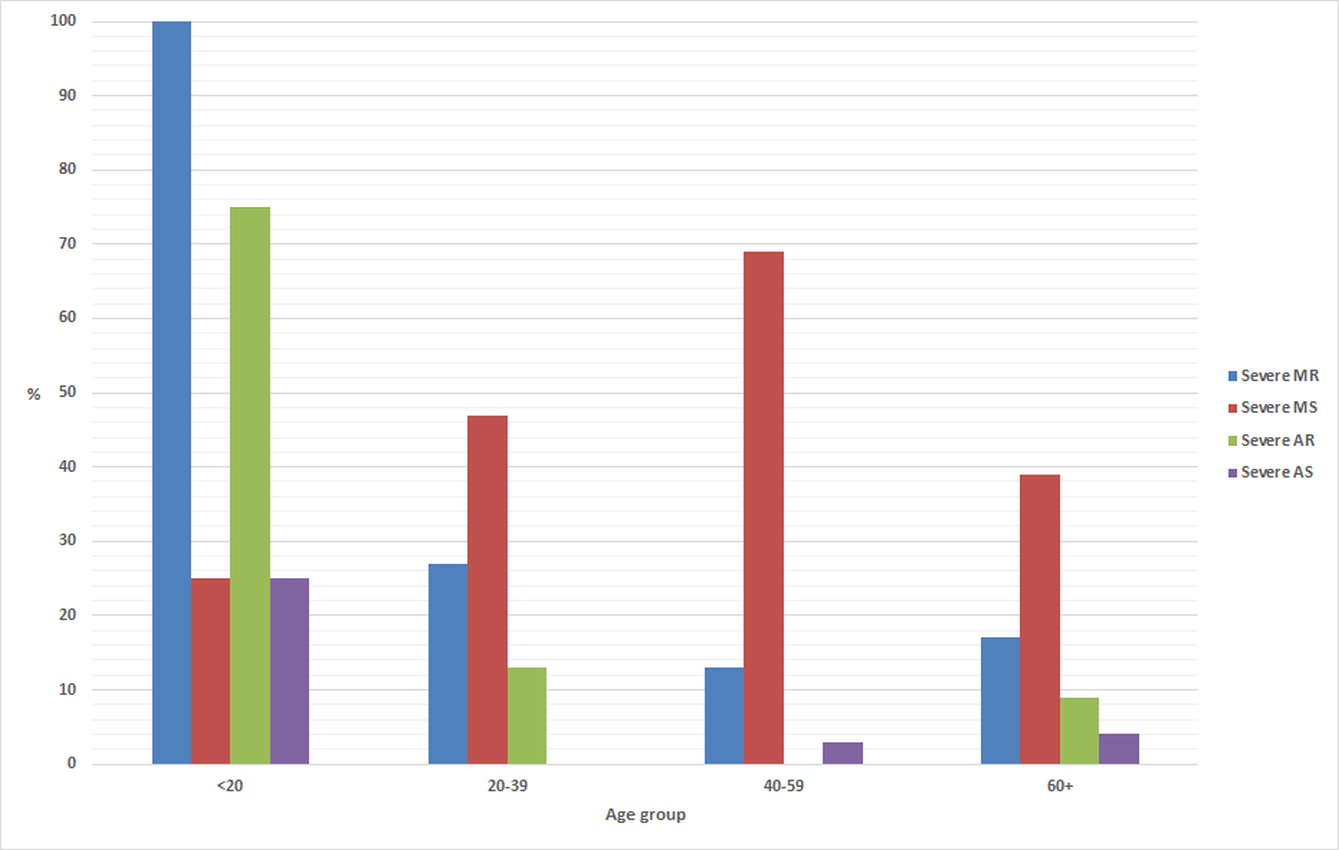
Pattern of valve involvement by age in the cohort.

Of the 96 patients, 92 (96%) had mitral valve involvement; in 38 (41%) the aortic valve was also involved, while in 54 (59%) the mitral valve was involved in isolation. Only 4 (4%) patients had the aortic valve in isolation. Rheumatic involvement of the pulmonary or tricuspid valve was not reported in any patient. Of the 92 patients with mitral valve involvement, 67 (73%) had severe mitral stenosis, severe mitral regurgitation or both. A Wilkins score was calculated in 22 patients: the median (range) score was 11 (7–13). Of the 42 patients with aortic valve involvement, 10 (24%) had severe aortic stenosis, severe aortic regurgitation or both. Overall 71 (74%) of the patients had severe valvular dysfunction and 79 (82%) met criteria for surgery or intervention. The other echocardiographic features are presented in Table 3 with the pattern of valve involvement by age presented in Fig 3.

### Management

Patients received diuretic therapy and, where indicated, negative chronotropic therapy. The single patient with suspected endocarditis received empirical antibiotic therapy (based on clinical findings and a mobile vegetation on echocardiography; facilities for blood cultures were unavailable). Only 32 (53%) of the 60 patients known to be in atrial fibrillation or flutter were discharged on anti-coagulation, this was usually due to the patients’ inability to afford the cost of INR monitoring. All 96 patients were discharged on long-term penicillin. Of the 41 female patients younger than 50 years of age, 14 (34%) were prescribed contraception on discharge. There were four patients referred for cardiology follow up and four patients referred for cardiothoracic surgical opinion. All the remaining patients had planned review in the specialist medical outpatients’ department of the hospital.

### Outcome and follow up

In the 12 months’ study period, three (3%) of the patients died. The first a 23-year-old woman with severe mitral regurgitation and aortic regurgitation died from cardiogenic shock during her second admission of the study period; the second, a 58-year-old man with moderate mitral regurgitation died in cardiogenic shock during his second admission of the study period. The third, a 60-year-old woman with severe mitral stenosis died suddenly at home one month after discharge.

Twenty-three of the patients were readmitted during the study period; 16 were readmitted once, three were admitted twice, three were readmitted three times and one was readmitted on five occasions. After a median (IQR) of 298 (161–348) days of follow-up, 28 (29%) had been lost to follow-up. Among the 65 who were alive in follow-up, 21 (33%) had ongoing symptoms related to their RHD. None of the patients referred for a surgical opinion had received surgical intervention during follow-up.

## Discussion

This prospective clinical audit of adult medical admissions to a typical general hospital in Myanmar, confirms the significant—and largely unmet—burden of RHD in the country. The patients in this cohort were predominantly young adults with advanced RHD who were commonly neither receiving recommended secondary prophylaxis nor appropriate medical treatment. Even after institution of medical therapy, ongoing disability and rehospitalisation was common, emphasising the challenges of caring for patients with RHD where there is no access to appropriate surgical care. The number of RHD presentations during the study period was over six times that of malaria admissions and a third the rate of HIV-related hospitalisations. However, unlike malaria and HIV, RHD receives very little funding in Myanmar’s health budget and lacks an integrated national programme to co-ordinate the multifaceted care that is necessary to reduce the burden of this preventable disease.

There is little doubt that this study significantly underestimates Myanmar’s true RHD burden. Patients were identified through clinical examination alone and it is recognised that for every clinically recognised case of RHD, there are up to ten subclinical cases [21, 22]. This is an important caveat, as untreated patients with subclinical RHD have a significant risk of developing symptomatic disease [23, 24]. Furthermore, by recruiting only subjects with clinical signs in a tertiary referral hospital setting, the study is less likely to identify patients with milder disease, a fact supported by the observation that over 80% of the cohort met criteria for surgery or intervention. Finally, RHD is a disease of poverty [25] and it is sobering to reflect that this study was performed in Yangon, one of the wealthiest regions in the country [26]. It would be anticipated that disease prevalence in other regions would be even greater and access to care far more limited [8, 27, 28].

The cohort’s annual mortality of 3.1% is within the range of 3.0–12.5% that is usually reported for RHD from low and middle-income countries [29], although this is likely to be an underestimate given the study’s loss to follow-up of almost 30%. Meanwhile, the important complications of advanced RHD, namely atrial fibrillation, cardiac failure, stroke and pregnancy-related complications were all seen commonly. As in other studies, the burden of disease fell predominantly on younger people with the majority of patients in the cohort younger than 50 years of age [30], while all of the deaths and almost three quarters of the strokes occurred in patients 60 years of age or younger [1, 31].

The very high rate of atrial fibrillation (63%) in the cohort was especially notable [32] and even this elevated figure is likely to be an underestimate given the high rates of subclinical atrial fibrillation seen in RHD patients [33]. The frequency of atrial fibrillation is particularly significant given the poor prognosis associated with this complication [34, 35] and–along with the limited access to anti-coagulation—is almost certainly responsible for the high rate of disabling stroke seen in the cohort [36]. The rate of significant obstetric complications was also high: 17% of all the women in the study had experienced a significant obstetric complication in a country where many women are unable to access optimal antenatal and reproductive health services [37, 38]. Infective endocarditis was also seen, although it is an especially challenging condition to diagnose and manage in a public health system which has only limited laboratory diagnostic support [39].

The delivery of health care in Myanmar remains fragmented and poor co-ordination of primary and specialist health services frequently results in patients failing to receive relatively simple, inexpensive interventions that would be expected to improve symptoms and reduce hospitalisations [40]. In this study over half of the cohort had a previous RHD-related hospitalisation, but 45% of these patients were not taking any medication at the time of their admission. Meanwhile, less than 20% of the patients with prior cerebrovascular event were on anti-coagulation, only 20% of women of child bearing age were receiving contraception and only 23% of patients with a strong indication for penicillin were receiving it [41]. More than half of the cohort had poor dentition, increasing their risk of infective endocarditis [42].

With the obvious challenges of delivering effective health care to patients with advanced RHD in a resource-limited setting like Myanmar, the focus must be on how to prevent the condition [43]. Secondary prevention–the regular prophylactic use of penicillin to reduce the risk of recurrent ARF and progression of RHD–is the most cost-effective strategy to prevent the disease and the easiest to implement [22, 44]. Secondary prevention requires case finding, referral, registration, administration of penicillin and regular follow-up and is most efficiently delivered within programmes that utilise a community-based registry [45]. A community based strategy has already been shown to be highly effective in diseases such as malaria and maternal and child health in Myanmar [8, 46, 47] and might be expected to be successful in RHD as well [48, 49]. Integration of an RHD programme with existing primary-care networks would be expected to defray the costs of such an approach [50]. This study’s high rate of loss to follow-up demonstrates the limitations of a centralised model of care, although these issues are not unique to Myanmar [43, 51].

Earlier recognition and initiation of therapy would be expected to improve outcomes. It is notable that even in the context of a clinical study in a tertiary referral hospital in the country’s largest city, almost 20% of the patients with clinical signs suspicious for RHD were unable to have an echocardiogram. Greater access to echocardiography would facilitate not only the diagnosis of RHD but would also assist the management of other cardiac diseases and has an established role in the management of non-cardiac disease [52]. While the costs of unrestricted echocardiography services are prohibitive, other strategies including the use of handheld devices may be a useful interim measure until, with the evolving health spending in the country, an expanded echocardiography service is feasible [40, 53].

The limited access to surgical care in the cohort is also striking. Out-of-pocket expenses for valve replacement in Myanmar’s private health system are approximately USD8000, while even at government hospitals out-of-pocket expenses are USD4000, meaning that surgery is out of the reach of almost every Myanmar citizen (personal communication, Professor Mar Mar Kyi). Although PBMV is less expensive (out-of-pocket expenses: USD1000) a benefit from this procedure is frequently short-lived [54]. It is also notable that with a median Wilkins echocardiographic score of eleven, PBMV in the cohort would be less successful [19, 35]. While analysis of the incremental cost-effectiveness suggested that building up valve surgery services was not cost-effective in Africa [44], this analysis did not appear to consider the potential for augmented cardiothoracic surgery services to improve the care of patients with other health conditions. Indeed, in Myanmar, ischaemic heart disease, tuberculosis and lung cancer are the second, sixth and ninth commonest causes of death in the country respectively, and all may be expected to benefit from improved access to cardiothoracic surgical care [9].

Myanmar is not the only country struggling with RHD, a disease of poverty associated with overcrowding, poor sanitation, and other social determinants of poor health [1]. Although the disease’s global prevalence is declining slightly, it remains relatively neglected when compared to many of the other diseases that affect these populations [3]. Global mortality due to HIV, tuberculosis and malaria is up to five times that due to rheumatic heart disease [55], but the expenditure on research and development for these diseases is 500 to 1000 times that spent on RHD [56].

So, what are the solutions? In 2015, a gathering of RHD experts in Addis Ababa released a communiqué that addressed strategies to eradicate the disease in Africa [57]. It is notable that many of the problems highlighted in that document–a lack of RHD surveillance, low utilisation of reproductive health services, centralisation of health services for RHD diagnosis and treatment, poor access to surgical services and an absence of national multi-sectoral initiatives on RHD prevention–were all seen in this Asian cohort. However, many of the potential solutions offered in the Addis Ababa communiqué might be also relevant to Myanmar. These include the decentralisation of the services used to diagnosis of RHD, the creation of prospective patient registries to quantify disease burden and track the progress of programme initiatives, improved access to reproductive health services for women with RHD, the development of international partnerships to mobilise resources and build capacity, the establishment of centres of excellence for essential percutaneous and surgical care of RHD and finally, the establishment of a national RHD programme to coordinate the cross-sectoral cooperation required to address disease comprehensively [57].

## Conclusions

A significant increase in funding in Myanmar has resulted in impressive recent progress against diseases as different as malaria, tuberculosis and HIV, but this success would not have possible without government-sponsored national programmes coordinating the implementation of effective disease control strategies. The successes of these ambitious national infectious diseases programmes show what can be achieved in Myanmar with sustained financial, political and scientific commitment. If an adequately funded national RHD programme were established in Myanmar, there should be no reason that these principles cannot also be applied successfully to address the significant burden of RHD as well.

## Supporting information

S1 Dataset RHD Finalized DATA for PLoS One.xlsx. (XLSX)
